# Traditional Chinese medicine promotes the control and treatment of dementia

**DOI:** 10.3389/fphar.2022.1015966

**Published:** 2022-10-11

**Authors:** Pengyu Tao, Wenxin Xu, Simeng Gu, Haiyan Shi, Qian Wang, Yuzhen Xu

**Affiliations:** ^1^ Department of Nephrology Seventh People’s Hospital Affiliated to Shanghai University of Traditional Chinese Medicine, Shanghai, China; ^2^ Department of Urology, Renji Hospital, Shanghai Jiao Tong University School of Medicine, Shanghai, China; ^3^ Department of Psychology, Jiangsu University Medical School, Zhenjiang, China; ^4^ Department of Social Health Management, Pingshan General Hospital of Southern Medical University, Shenzhen, China; ^5^ Department of Central Laboratory, The Affiliated Taian City Central Hospital, Qingdao University, Qingdao, China; ^6^ Department of Rehabilitation, The Second Affiliated Hospital of Shandong First Medical University, Taian, China

**Keywords:** tau protein, Chinese herbal medicine, dementia, oxidative stress, inflamation

## Abstract

Dementia is a syndrome that impairs learning and memory. To date, there is no effective therapy for dementia. Current prescription drugs, such as cholinesterase inhibitors, fail to improve the condition of dementia and are often accompanied by severe adverse effects. In recent years, the number of studies into the use of traditional Chinese medicine (TCM) for dementia treatment has increased, revealing a formula that could significantly improve memory and cognitive dysfunctions in animal models. TCM showed fewer adverse effects, lower costs, and improved suitability for long-term use compared with currently prescribed drugs. Due to the complexity of ingredients and variations in bioactivity of herbal medicines, the multi-target nature of the traditional Chinese formula affected the outcome of dementia therapy. Innovations in TCM will create a platform for the development of new drugs for the prevention and treatment of dementia, further strengthening and enhancing the current influence of TCM.

## Introduction

Dementia is associated with progressive impairments in memory and learning abilities, cognitive skills, behavior, daily-living activities, and quality of life (McShane et al., 2019; Zhang et al., 2022). More than 47.5 million people worldwide are affected by dementia, and 7.7 million new cases are added each year to the dementia pool (Padilla, 2019; Wang et al., 2020a). The most common type of dementia is Alzheimer’s disease (AD), accounting for approximately 70% of the total dementia cases (Babulal et al., 2019). The second most common cause is vascular dementia (VaD), constituting 10%–15% of the total number (Xu et al., 2017). The latest advances in medical technology, such as optogenetics, provide us with novel insights into the mechanism of dementia (Chen W. et al., 2022). AD often coexists with VaD, the most common type of mixed dementia. Currently, only six drugs, including four cholinesterase inhibitors, one N-methyl-d-aspartate (NMDA) receptor antagonist, and one monoclonal antibody against A-beta, have been approved by the FDA for the treatment of AD (Xu et al., 2016; Zhang J. et al., 2020). However, these drugs only slightly improve cognitive function, with no effect on prolonged survival rate. Some anti-AD drugs such as donepezil and galantamine are used off-label for the treatment of VaD and have shown moderate improvements in cognitive function. However, none of these drugs are sufficiently effective (Wang et al., 2020b). Hence, due to the absence of satisfactory pharmacological therapies, the focus on herbal medicine has increased, to treat AD and VaD in new ways. Chinese herbal medicine has long been used to improve memory (Hui et al., 2010), suggesting that it has potential in the treatment of dementia. The current study reviews some updated advances in pre-clinical and clinical research to support the use of traditional Chinese medicine (TCM) for the treatment of dementia, particularly AD and VaD, and discusses the challenges and debates.

## The role of protein aggregation in dementia

Phosphorylated tau protein and amyloid-β are two types of protein aggregations (Cai et al., 2021). Amyloid precursor protein (APP) is a transmembrane protein (Abdelnour et al., 2020). The three key secretory enzymes, α, β, and γ, split and release the APP component into the cytoplasm (Branton, 2021). Under normal circumstances, *a* and γ secretory enzymes cleave APP to form β 17–40/42 (Frank et al., 2020). However, in patients with AD it was shown that β secretory enzyme possibly cleaves the APP to form extracellular, soluble APP-β (Goulay et al., 2020). Subsequently, γ secretory enzymes cleave the APP-β to form amyloid-β 1–40/42 and release it into the extracellular environment, where it is later degraded by a protease (Munasinghe et al., 2020). Soluble amyloid-β plays a beneficial role in memory and synaptic transmissions among cells (Lee et al., 2021). However, amyloid-β aggregations form insoluble protein fibers and promote amyloid plaque depositions, leading to cell death (Zhang et al., 2021).

Tau is a microtubule-binding protein widely found in nerve cells. The aggregation of tau protein is a critical indicator in neurodegenerative diseases (Spillantini et al., 1998). Tau is a specific neuron microtubule-associated protein that regulates the stability of microtubules and is thereby necessary for the formation of axons and synaptic plasticity of nerve cells (Kopach et al., 2020). However, under pathological conditions tau protein forms neurofibrillary tangle (NFT). Since the discovery of AD, tau protein aggregation has been a crucial criterion in the diagnosis of AD (Carlomagno et al., 2021). Three forms of aggregation involve the tau protein: NFT in neuronal soma, fibrous webs in dendrites, and plaques (Mochizuki et al., 2011). The tau protein interferes with the normal activities of nerve cells in various ways. First, tau protein aggregation interferes with the protein’s normal function, reducing microtubule stability (Schillaci et al., 2021; Silva et al., 2021). Some studies have reported that phosphorylated tau protein depolymerizes microtubules, affecting the transport of axonal materials (Schillaci et al., 2021). Second, the NFT formed by the tau protein aggregation may be toxic to cells, leading to a low survival rate of neurons.

The concentration of tau protein aggregations in nerve cells is positively correlated with AD progression. Hence, numerous studies have been performed to clarify the role tau protein plays in AD, to develop novel drugs that can reverse or eliminate tau protein aggregation. Tau is an inherently disordered protein, comprising 441 amino acids. Phosphorylated tau protein has been detected in the hexapeptide repeat and proline-rich regions of patients with AD.

**FIGURE 1:**
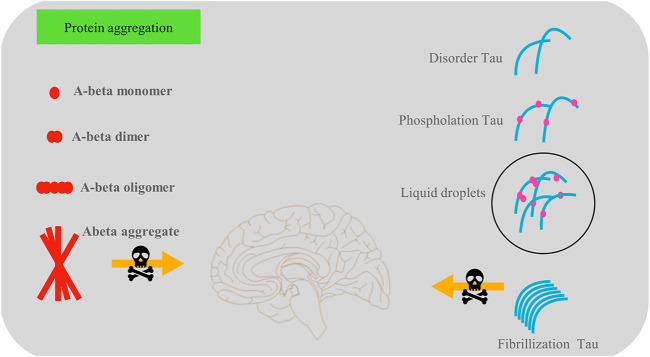
There are two main types of protein aggregation—phosphorylated tau protein and amyloid-β protein aggregations, which play a vital role in the development of dementia. Soluble amyloid-β is beneficial in memory and synaptic transmissions among cells, but amyloid-β aggregations are harmful to cell survival. The tau protein interferes with the normal activities of nerve cells in a variety of ways, such as by reducing microtubule stability.

Recent studies have reported that phase separation initiates the transformation of tau protein aggregation into NFTs and that phosphorylation makes tau more prone to phase separation. Non-bond interaction is one of the primary driving forces regulating the phase separation process. Some small molecule drugs, such as phenothiazine or triarylmethane derivatives, appear to have potential in inhibiting tau protein aggregation via non-covalent interactions. These molecules have a centrosymmetric structure and delocalized positive charge that integrates with the π electron cloud of aromatic amino acid side chains through van der Waals forces. However, these drugs often have no specificity, and their effectiveness requires further exploration. The role of protein aggregation in the progression of dementia is shown in Figure 1.

## The role of oxidative stress and inflammation in dementia

### Oxidative stress in dementia

Superoxide dismutase (SOD), GPX, and cytochrome c are important antioxidants that under normal circumstances clear redundant reactive oxygen species (ROS) (Papandreou et al., 2008). Various studies have reported that the antioxidant system is abnormal in patients with dementia, contributing to oxidative stress (Bennett et al., 2013; Xu et al., 2021). The concentration of cytochrome c in patients with AD was 20%–30% lower than that in people without AD. A high level of activity of SOD was detected in the CA1 region of the hippocampus in patients with AD (Han et al., 2012). In a transgenic mouse AD model, abnormal mitochondrial transport was observed during the embryonic stage, and the division and fusion of mitochondria were also different from those seen in normal mice. This increase in ROS production due to mitochondrial abnormalities has been observed in many human patients and in animal models, prior to tau protein or amyloid-β aggregation.

The hemostasis of metal ions is closely related to the production of ROS (Wang and Wang, 2017). The accumulation of metal ions generally occurs in the amyloid plaques in the brains of patients with AD (Sensi, 2014). Metal ions such as copper and zinc reportedly produce free radicals in the brain via the Fenton reaction, leading to metal ion-related oxidative stress in the brain. Furthermore, amyloid-β increases the production of ROS by combining with metal ions (Gomes et al., 2020; Rychlik and Mlyniec, 2020). However, metal chelating agents reduce amyloid plaques in the cerebellum and improve cognitive function. Increased oxidative stress and abnormal antioxidant pathways play a role in the pathogenesis of AD. This is associated with amyloid-β and tau protein interactions, forming a vicious cycle that leads to the death of neurons.

Hypoxia-induced oxidative stress is possibly associated with mitochondrial abnormalities, neuronal inflammation, and apoptosis (Vinters, 2004). Oxidative stress disrupts the balance between antioxidants and ROS elimination, resulting in vascular endothelial cell, glial cell, and nerve cell damage (Ryan et al., 2005). In addition, hypoxia releases inflammatory factors into blood vessels. Some inflammatory factors, such as matrix metalloproteinases, weaken the protective effect of the blood–brain barrier (Thompson and Lennox, 2005). Increased blood–brain barrier permeability allows the influx of more inflammatory factors, including IL1, IL6, and TNF-α, into the brain (Clionsky and Clionsky, 2012). Once these inflammatory factors accumulate in the brain, adverse effects are observed in the white matter (neuron demyelination and axon loss), and there is apoptosis of neurons. The inflammatory cascade in the hippocampus inhibits nerve regeneration, neural progenitor cell proliferation, and synaptic remodeling, resulting in a decline in memory and cognitive function (Ren et al., 2019).

### Inflammation in dementia

Inflammation and dementia have a close link (Xu et al., 2019; Chen Y. et al., 2022). An appropriate inflammation response helps repair tissues by clearing hazardous substances, such as bacteria and viruses (Hermida et al., 2012), but sustained inflammation does more harm than good to tissues. It aggravates the damage to the integrity of nerve fiber sheath by intensifying the inflammatory reaction or oxidative stress disorder, thereby worsening dementia (Hermida et al., 2012). Inflammatory markers, including C-reactive protein, antitrypsin, interleukin (IL), and homocysteine, can be used to detect dementia (Enciu and Popescu, 2013). Overexpression of IL-1 initiates the mechanism of inflammation that affects dementia (Yasutake et al., 2006). IL-1 affects tau protein aggregation by regulating APP and amyloid-β generation and accompanies the increased expression of IL-6 and tumor necrosis factor α (TNF-α) (Rainero et al., 2004). IL-1 also increases the sensitivity of neurons to the inflammatory response by increasing the uptake of Ca^2+^ through the NMDA receptor ion channels, further aggravating dementia (Yucesoy et al., 2006). Moreover, the IL-1-mediated NF-кB pathway is another vital inflammatory signaling pathway in dementia (Guo et al., 2018). IκB inhibits NF-кB activity. The overexpression of IL-1 activates IκB kinase complex. Subsequently, IκB is phosphorylated and degraded (Atasoy et al., 2015). Translocation of NF-кB into the nucleus immediately after the inhibition effect of IκB is halted. Lastly, NF-кB in the nucleus promotes gene transcription, leading to neuronal inflammation and apoptosis (Forlenza et al., 2009).

IL-6 is secreted by monocytes and is maintained at a relatively low level in the normal physiological state (Park et al., 2010). Various pathological factors increase IL-6 levels, including senescence, hypertension, and obesity. The function of IL-6 in the nervous system mainly involves three aspects: ① IL-6 promotes neuron growth and differentiation by stimulating the activation of the neuroendocrine system; ② IL-6 cooperates with IL-1 and TNF-α to enhance the inflammatory response; ③ IL-6 may be closely associated with the abnormal phosphorylation of tau protein (Zhu et al., 2005). Generally, IL-6 intensifies oxidative stress damage to brain tissue by enhancing the expression of pro-inflammatory cytokines in downstream signaling pathways, leading to neuron loss or apoptosis. Reportedly, the overexpression of IL-6 promotes amyloid-β deposition, affecting the integrity of neuronal axons and dendritic membranes (Mausbach et al., 2019). The increased expression of inflammatory factors such as IL-6 and IL-1 in the brain enhances the inflammatory response and oxidative stress disorder, depositing twisted proteins and promoting neuronal apoptosis (Uslu et al., 2012). In addition, the synergistic effect of the two inflammatory factors enhances phagocytosis by natural killer (NK) or glial cells and leads to the decline of neuronal function in patients with AD (Luigi et al., 2001). Studies have shown that IL-1 and IL-6 levels are significantly positively correlated with MMSE scores in patients with dementia, suggesting that IL-1and IL-6 are closely associated with the development of dementia (Wada-Isoe et al., 2010).

**FIGURE 2:**
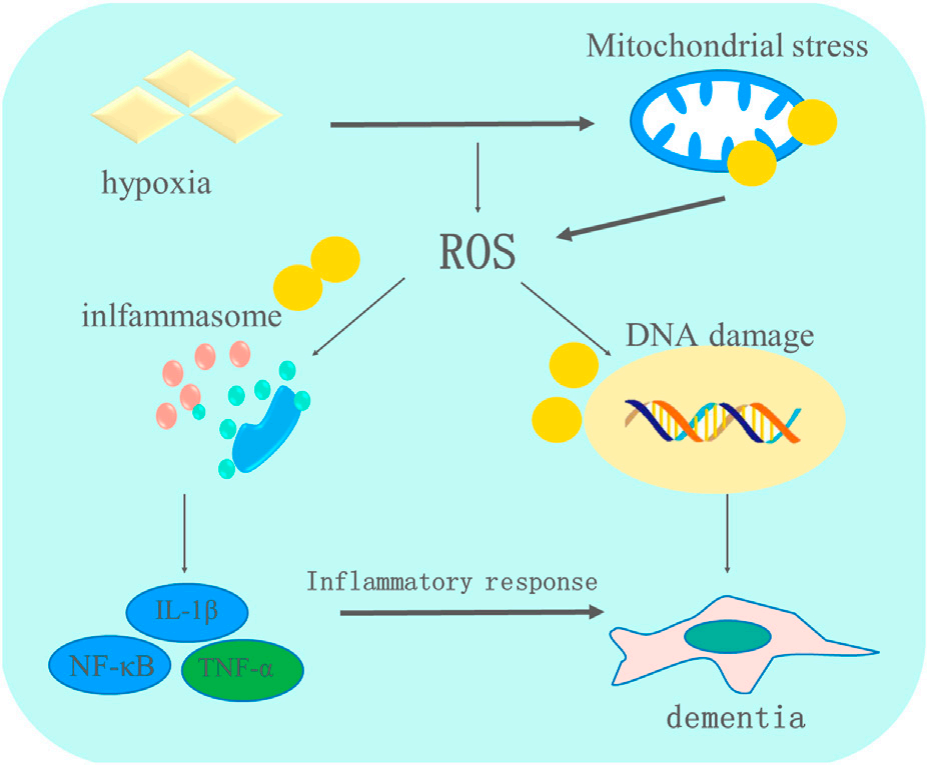
The role of oxidative stress and inflammation in the progression of dementia. Multiple pathological factors contribute to the occurrence of dementia. Hypoxia-induced oxidative stress impairs mitochondria, promoting the generation of ROS, which further stimulates the activation of inflammasomes that relieve pro-inflammatory cytokines and finally leads to the apoptosis of brain cells, which aggravates the condition of dementia.

TGF-β is a multifunctional protein that regulates cellular growth, differentiation, apoptosis, and immunomodulation (Moses, 2010). TGF-β serves a dual role in the human body. High levels of TGF-β expression increase exogenous glutamate production, which contributes to neuronal damage by suppressing the activation of glutamine synthetase in astrocytes (Carta et al., 2017). A study reported that high TGF-β expression during the chronic activation of microglia in brain tissue may have resulted in nerve damage by releasing potentially cytotoxic molecules. The underlying mechanism may be that the upregulated TGF-β promoted the generation of astrocytes, contributing to cognitive impairment through the angiotensin-type 1 receptor-mediated signaling pathway, and an angiotensin-converting enzyme inhibitor may reverse the adverse effect of TGF-β in astrocytes (Ongali et al., 2018). These results suggest that TGF-β as an inflammatory factor enhances the inflammatory response during the progression of dementia. However, TGF-β secreted by astrocytes showed protective effects against brain injury caused by ischemia (Zhao et al., 2020). TGF-β exerted anti-inflammatory effects by inhibiting the proliferation and activation of microglia and astrocytes. The mechanism behind these may be related to the inhibited role of TGF-β in exerting neuroprotective effect against dementia through suppressing the expression of some inflammatory protein expressions (Chen et al., 2015). In addition, the TGF-β level was shown to be significantly increased in patients with dementia compared with a healthy control group, indicating that TGF-β may have a pro-inflammatory role (Tarkowski et al., 2002). According to some researchers, TGF-β could be a new therapeutic target to inhibit neuro-inflammatory reactions, reducing dementia-related neurodegenerative disease (Chen et al., 2015). The role of oxidative stress and inflammation in dementia is shown in Figure 2.

## Traditional Chinese formulations for dementia

Recently, the role of TCM in treating dementia has gained attention. Several studies have shown an effect of herbal formulations on the prevention of dementia and are summarized in Table 1.

**TABLE 1:** Herbal formulas that exert protective effects against dementia.

Herb formula	Protective mechanism
Suan Zao Ren decoction	Anti-inflammation and oxidation protects against dementia through Axl/HSP 90/PPARγ pathway activation (Mu et al., 2018)
Buyang Huanwu decoction	Protects the integrity of synaptic structures by blocking the expression of SYN (SYN-1), GAP-43 and MAP-2 (Cui et al., 2015)
Taohong Siwu decoction	Promotes angiogenesis to restore blood circulation by upregulation of VEGF (Lan et al., 2015)
Lingyang Gouteng decoction	Attenuates oxidative stress damage by restoring SOD3 activation and inhibition of NOX2 expression (Zhao et al., 2018)
Huanglian Jiedu decoction	Improves sphingolipid metabolism by prohibiting SGMS1 and enhancing CERS2 expression (Chiang et al., 2021)

The data shown in Table 1 suggest that traditional Chinese decoctions can exert neuronal protection against dementia through activating or inhibiting the expression of various transduction signal pathways.

### Suan Zao Ren decoction

Suan Zao Ren decoction (SZRD) is a widely used mixture consisting of five Chinese herbs, including *Ziziphi spinosae* Semen, *Poria*, (something) Rhizoma, *Anemarrhenae* Rhizoma, and Glycyrrhizae Radix Et Praeparata Cum Melle. This treatment was created by Zhang Zhongjing, a medical expert who lived during the Han dynasty, and was generally used to relieve uneasiness of the mind by nourishing the yin and the blood. Studies have found that SZRD may delay the development of dementia by improving cognitive impairment and repairing neuron damage (Long et al., 2021). These results were confirmed in an animal model of dementia. Some key extracts from SZRD were identified in an attempt to understand its mechanism in treating dementia. Jujuboside A is the most studied extract that exerts anti-inflammatory effects and oxidation against dementia, via the activation of the Axl/HSP 90/PPARγ pathway (Mu et al., 2018). Spinosin and mangiferin showed similar efficacy to Jujuboside A; however, spinosin reduced oxidative stress in dementia (Zhang X. et al., 2020), while mangiferin attenuated synapse damage by inhibiting tau protein phosphorylation (Du et al., 2019). Clinical trials and animal studies have confirmed the role played by SZRD in the treatment of dementia.

### Buyang Huanwu decoction

Buyang Huanwu decoction (BYHWD), which consists of Radix Astragali, Radix Angelicae Sinensis, Radix Paeoniae Rubra, Chuanxiong Rhizoma, Semen Persicae, Flos Carthami, and Pheretima, has been used in China for centuries to help recover neurological function in brain injury cases. Cerebral ischemia may play a key role in vascular dementia (Li et al., 2013). BYHWD could assist in the recovery from this via neuronal plasticity and remodeling. An investigation into BYHWD showed that it protects the neurons located in the hippocampal CA1 region from apoptosis by suppressing the expression of caspase-3 (Luo et al., 2017). Another study reported that BYHWD, combined with physical exercise, in a 14-day treatment of animals with cerebral ischemia protected the integrity of the synaptic structure by reducing the protein levels of SYN (SYN-1), GAP-43, and MAP-2, which are used as biomarkers of neuro-rehabilitation (Cui et al., 2015). Plaque deposits of amyloid-β are one of the main characteristics of dementia. The receptor for advanced glycation end-products (RAGE) and lipoprotein receptor-related protein1 (LRP1) are vital carriers for the transfer of Aβ to the brain, by crossing the blood–brain barrier. However, this translocation could be inhibited by BYHWD, thereby reducing RAGE and LRP1 levels in the brain (Liu et al., 2019). Studies have shown that the protective mechanism of BYHWD mainly relies on inhibiting neuron apoptosis, promoting neurorehabilitation, and suspending Aβ translocation across the blood–brain barrier.

### Taohong Siwu decoction

The core use of Taohong Siwu decoction is promoting blood circulation and removing blood stasis; in China, it is often used to treat women who have irregular menstruation. This famous formula originated from “YiZongJinJian,” a classic text in Chinese medicine. It is composed of *Rehmannia glutinosa* Libosch, *Paeonia lactiflora* Pallas, *Angelica sinensis*, *Ligusticum chuanxiong* Hort, *Prunus persica*, and *Carthamus tinctorius* L. Based on the “new blood generated by removing blood stasis” theory, Taohong Siwu decoction is effective in alleviating vascular-related neurological diseases, such as vascular dementia. Although the chemical components of Taohong Siwu decoction are complex, its longstanding use can guarantee its safety and efficacy. One study reported that Taohong Siwu decoction attenuated cerebral ischemia by promoting angiogenesis and restoring blood circulation. The underlying molecular mechanism included the upregulation of vascular endothelial growth factor, which is vital for angiogenesis, followed by an increase in the protein level of CD34, which is a positive regulator in neurogenesis.

Memory dysfunction is closely associated with the elderly population with vascular dementia or pathological cerebral changes, for which ischemia is a critical cause. A behavior test showed that treatment with Taohong Siwu decoction greatly improved the impairment of memory and learning undergone by animal models with vascular dementia (Lan et al., 2015). With the upregulation of Bcl-2 expression and downregulation of Bax expression (these two proteins exert anti-apoptotic and pro-apoptotic effects, respectively) (Gai et al., 2010), more neurons survived in the hippocampal CA1 region following the administration of Taohong Siwu decoction (Jinglong et al., 2013). These results indicate that Taohong Siwu decoction shows promising efficacy in neuroprotection.

### Lingyang Gouteng decoction

In TCM, an imbalance between *Qi* and the blood due to the hyperactivity of liver yang is associated with the occurrence of dementia characterized by memory loss, decreased learning function, and social obstacles. Lingyang Gouteng decoction (LYGTD) is a classic treatment that inhibits hyperactivity of the liver yang-related syndrome; it comprises *Uncaria rhynchophylla* (Gou teng), *Morus alba* L (Sang ye), *Fritillaria cirrhosa* (Bei mu), *Bambusa tuldoides* Munro (Ba ji), *Rehmannia glutinosa* Libosch (Di huang), *Chrysanthemum morifolium* Ramat (Ju hua), *Paeonia lactiflora* Pall (Shao yao), Poria cum Radix Pini (Fu ling), and *Glycyrrhiza uralensis* Fisch (Gan cao). In addition to cerebral ischemia, oxidative stress contributes to the development of vascular dementia (Hoyos et al., 2022). SOD3 is a strong antioxidant enzyme suppressed during oxidative stress, and nicotinamide adenine dinucleotide phosphate oxidase 2 (NOX2) is closely related to oxidative stress damage (Bennett et al., 2013). LYGTD reportedly exhibited strong neuroprotection against oxidative stress in vascular dementia by activating SOD3 to eliminate free radicals and inhibiting NOX2 expression to attenuate oxidative stress damage to neurons in the brain (Zhao et al., 2018). Furthermore, LYGTD effectively maintained neurovascular coupling sensitivity, a crucial function maintaining sufficient blood to preserve brain function during cerebral ischemia (Zhao et al., 2018). Hence, LYGTD possibly targets oxidative stress in vascular dementia.

### Huanglian Jiedu decoction

Huanglian Jiedu decoction is generally used for clearing “heat” and removing toxic substances; in Chinese medicine, “heat” could be interpreted as inflammation (Chiang et al., 2021). Huanglian Jiedu decoction is composed of *Coptis chinensis* Franch, *Scutellaria baicalensis* Georgi, *Phellodendron amurense* Rupr, and *Gardenia jasminoides* Ellis. Chinese medicine theory focused on the role of “toxicity” in damaging brain function and became the guideline for the diagnosis and treatment of brain diseases for centuries. Research into this theory has identified that “toxicity” referred to amyloid-β accumulation in the brain (Liu et al., 2017). Sphingolipids, vital lipids located on the cell membrane, promote the take up of amyloid-β by microglial cells (Vetrivel and Thinakaran, 2010). However, sphingolipid metabolism is interrupted in AD, leading to the overgeneration and accumulation of amyloid-β. A study showed that Huanglian Jiedu decoction could protect sphingolipid metabolism from disturbance by prohibiting SGMS1 and enhancing CERS2 expression. The interaction between monocytes and microglia is a protective mechanism to prevent amyloid-β from passing through the blood–brain barrier. Inflammatory cytokines deactivate this protective mechanism, leading to amyloid-β accumulation in the brain (Fisher, 2021). Administration of Huanglian Jiedu decoction could reverse this unfavorable situation by relieving the inflammatory factors, such as IL-6 and INF-γ.

## Conclusion

The efficacy of the classic Chinese herb formula for the treatment of dementia has been confirmed by performing intensive clinical trials and scientific studies. These traditional decoctions were first used in people who were experiencing anxiety, anger, and insomnia, as at the time people did not develop dementia due to their shorter lifespan than that of modern people. However, the scope of application of these traditional decoctions can be expanded in later life. Multiple experiments have confirmed that these classic Chinese decoctions exhibit considerable potential for treating dementia. The protective mechanisms of these formulas are associated with promoting neuron survival, inhibiting apoptosis, reducing ROS generation, and blocking the release of inflammatory factors. However, these decoctions were not originally invented for people with dementia. Hence, more studies into the protective effects of these classic decoctions should be performed on the elderly population without dementia. Additionally, a standard quality control measure should be established for herb preparation, storage, and dosage, due to the complexity of the compounds.
